# Precision feeding gestating sows: effects on offspring growth performance and carcass and loin quality at slaughter

**DOI:** 10.1093/tas/txab227

**Published:** 2021-12-09

**Authors:** Lauren L Hansen, Victoria Stewart, Ira B Mandell, Lee-Anne Huber

**Affiliations:** Department of Animal Biosciences, University of Guelph, Guelph, Ontario, N1G 2W1Canada

**Keywords:** carcass quality, electronic sow feeder, gestating sows, loin quality, offspring, precision feeding

## Abstract

A total of 601 pigs from 65 litters were used to determine the effects of closely meeting estimated daily Lys and energy requirements for sows during gestation for three consecutive parities on offspring postweaning growth performance and carcass and loin quality at slaughter. Sows were assigned a control (static diet composition; **CON**) or precision (individual daily blend of two diets to meet estimated Lys and energy requirements; **PRE**) feeding program between days 7 and 110 of gestation for three consecutive pregnancy cycles, starting with primiparous sows (parity 1: 12 CON and 12 PRE sows; parity 2: 8 CON and 13 PRE sows; parity 3: 8 CON and 12 PRE sows). At weaning (20 ± 2 d of age), up to 10 pigs per litter were randomly selected and placed in a pen (1 litter per pen). All pens received ad libitum access to commercial diets in six phases (four-phase nursery, grower, and finisher, respectively). Four pigs per pen were slaughtered at ~125 kg BW for evaluation of carcass characteristics and loin quality. The ADG and ADFI of offspring were not influenced by maternal feeding program in any parity during nursery phases I through III. During nursery phase IV, ADG and ADFI were greater for litters from PRE- vs. CON-fed sows (0.70 vs. 0.66 ± 0.03 and 1.15 vs. 1.08 ± 0.06 kg/d for ADG and ADFI, respectively; *P* < 0.05). The BW for litters from PRE- vs. CON-fed sows tended to be greater by day 66 of age (end of nursery period; 29.7 vs. 28.7 ± 1.1 kg; *P* = 0.076). Within the grower phase, litters from PRE-fed sows had a greater ADG in parity 2 but lower ADG in parity 3 vs. litters from CON-fed sows (0.99 vs. 0.94 and 0.93 vs. 1.01 ± 0.03 kg/d for parities 2 and 3, respectively; *P* < 0.05). No differences were observed for ADG or ADFI in the finisher phase or G:F in any phase for any parity. Loin eye area was smaller (52.2 vs. 55.0 ± 1.8 cm^2^; *P* < 0.05) for offspring from PRE- vs. CON-fed sows. In parity 2, carcass lean yield tended to be less for offspring from PRE- vs. CON-fed sows (58.6 vs. 59.6 ± 0.4%; *P* = 0.051). Minimal differences were observed for subjective and objective evaluations of loin quality. Closely meeting the estimated daily energy and Lys requirements for sows throughout gestation for three consecutive pregnancy cycles improved offspring growth performance (ADG and ADFI) in the final nursery stage, but generally did not affect growth performance in grower/finisher periods or carcass and loin quality at ~125 kg BW.

## INTRODUCTION

Nutrient and energy requirements for sows during gestation and across parities are dynamic (NRC, 2012; Dourmad et al., 2017). In particular, the fetal pool undergoes significant changes in the rate of protein deposition, becoming exponential in the final trimester (McPherson et al., 2004; NRC, 2012). The acceleration of fetal protein deposition increases both amino acid (Lys) and energy requirements for the gestating sow, such that, estimated Lys and energy requirements for primiparous sows increase by ~200% and 45%, respectively, in late gestation compared with early gestation (NRC, 2012). By providing a static gestation diet in terms of quantity and nutrient composition, it is impossible to meet the nutrient and energy requirements for both maternal and pregnancy-associated protein deposition, which could have negative implications for fetal development (Hansen et al., 2014).

Previous studies have shown that in addition to negative effects on sow reproductive performance and longevity, postnatal growth performance for offspring can also be impacted when the maternal diet over/under supplies nutrients and(or) energy during gestation (Yang et al., 2009; Metges et al., 2014; Goncalves et al., 2016). Depending on the timing and extent of nutrient and(or) energy intake miss-match vs. requirements, the effects on the offspring can differ. For example, feeding a low-energy diet (4,417 kcal/d) in early gestation increased piglet weaning weights, while oversupplying energy during early gestation (10,229 kcal/d) reduced ADG and G:F for offspring in the growing–finishing period (Bee, 2004). Conversely, Vázquez-Gómez et al. (2018) found that feeding sows only 70% of the daily recommended requirements for energy in mid gestation (days 38 to 90) resulted in offspring with a lower ADG from birth until 215 d of age and reduced carcass yield at slaughter. Moreover, Chen et al. (2017) found that offspring from sows that were energy-restricted throughout gestation had lower relative small intestine weights combined with smaller villus height and crypt depth and lower BW at 28 d of age.

In most other stages of swine production, it is common to adjust diet composition in order to optimize growth rates, while minimizing feed costs. Yet, the practice of closely meeting both daily estimated energy and amino acid (Lys) requirements for sows throughout gestation has not been applied, and the implications on offspring performance outcomes until market weight have not been explored. It is hypothesized that closely matching daily estimated energy and Lys requirements of gestating sows over three consecutive parities will optimize offspring postweaning growth performance due to improved gastrointestinal tract development, which will maximize carcass and loin quality. Therefore, the objective of this study was to determine the effects of closely meeting the estimated daily energy and Lys requirements for individual sows during gestation over three consecutive parities on offspring post-weaning growth performance and carcass and loin quality at market weight (~125 kg BW).

## MATERIALS AND METHODS

The experimental protocol was approved by the University of Guelph Animal Care Committee and followed Canadian Council on Animal Care guidelines (CCAC, 2009). The study was conducted at the Arkell Swine Research Station, which is considered a high-health herd (OMAFRA, University of Guelph, Arkell, ON, Canada).

### Animals, experimental diets, and maternal feeding programs

One hundred five primiparous sows (62 Yorkshire and 43 Yorkshire × Landrace) were enrolled in the study and placed into one of four pens equipped with an electronic sow feeder (**ESF**; Canarm Agsystems; Arthur, ON, Canada) 5 ± 3 d after breeding over five consecutive breeding batches (blocks; one batch was bred per month). Sow genetics were distributed equally between treatments; sows were bred with either Yorkshire, Landrace, or Duroc semen. On day 110 ± 1 of gestation, sows were moved to individual farrowing crates. Within the first 24 h after farrowing, litters were standardized to between 10 and 12 piglets, depending on piglet availability among sows from the same maternal feeding program; litter characteristics at birth and piglet processing procedures are described by Stewart et al. (in press). Piglets received ad libitum access to a commercial creep feed (Floradale Feedmill Ltd., Floradale, ON, Canada; creep feed disappearance was not recorded) 7 d after birth and were weaned after a 20 ± 2-d lactation period. Sows were re-bred after 5±1 d and entered the same feeding program as in the previous pregnancy in the subsequent two reproductive cycles.

The ESF were supplied by two feed lines and had the ability to dispense precise amounts of each feed (Buis, 2016; Stewart et al., in press). The two basal diets were isocaloric (2,518 kcal/kg NE) but were formulated to contain high or low protein contents [0.80% and 0.20% standardized ileal digestible (**SID**) Lys, respectively]. In the first pregnancy cycle, each sow was randomly assigned to one of two gestation feeding programs: control (**CON**) or precision (**PRE**). The CON sows received a constant blend of 1.32 kg of the high protein diet and 0.88 kg of the low protein diet on each day of gestation to mimic a conventional industry feeding program (parity 1: *n* = 55; parity 2: *n* = 37; parity 3: *n* = 24). For the PRE feeding program, the NRC (2012) Nutrient Requirements Gestating Sow Model was used to estimate nutrient requirements on each day of gestation for each individual sow using body weight and reproductive cycle (parity; at the time of entry into group housing for each pregnancy) and estimated litter size (13.5) and piglet birth weight (1.4 kg; Buis, 2016; Stewart et al., in press). The model then determined the amount of the basal diets to blend together on each day of gestation to closely match the daily estimated Lys and energy requirements (parity 1: *n* = 50; parity 2: *n* = 36; parity 3: *n* = 25). The ESF dispensed the corresponding amounts of each diet. Upon entry to the farrowing crates, sows were fed 2 kg/d of a standard lactation diet (2520 kcal/kg NE and 0.74% SID Lys) until farrowing. Following farrowing, the amount of feed offered was gradually increased until day 5 of lactation where ad libitum feeding was achieved and maintained until weaning. Following weaning, sows entered the same gestation feeding program as in the previous pregnancy cycle.

### Offspring management and feeding

Litters containing at least 10 piglets were randomly selected from sows that began the study as primiparous sows, with the goal of obtaining 12 litters per gestation feeding program per parity. In other words, within parity, the number sows that had offspring subsampled depended on litter size and litters were not necessarily selected from the same sows across parities as some sows were removed due to lameness, reproductive failure, etc., as the study progressed (Stewart et al., in press). At weaning, up to10 and a minimum of 6 piglets per litter were selected to achieve an even sex distribution but were otherwise randomly selected. Thus, from a subset of 65 sows, a total of 601 piglets were selected over the three parities (litter was the experimental unit; parity 1: 12 CON and 12 PRE litters; parity 2: 8 CON and 13 PRE litters; parity 3: 8 CON and 12 PRE litters). At weaning, the piglets were placed in 1.3 × 3.0 m pens with plastic-coated expanded metal floors in an environmentally controlled nursery room (1 litter per pen). Each pen contained one stainless-steel feeder with four feeding spaces and water was freely available via two nipple drinkers. Two pigs per pen were removed on days 20 and 28 ± 2 of age for euthanasia with the goal of maintaining 6 pigs per pen between 28 d of age and market weight (i.e., ~125 kg BW) for growth performance outcomes. At 56 d of age, the pigs were moved to environmentally controlled grower-finisher pens (4.24 × 1.97 m; 1 litter per pen) that had partially slatted concrete floors. One stainless-steel feeder with three feeding spaces was provided per pen and water was freely available in a single bowl drinker.

Commercial nursery diets were provided in a four-phase feeding program with phases I, II, and III fed for 1 wk each, and phase IV fed for 24 days (micro pelleted for phase I, and pelleted for phases II, III, and IV; Table 1). On day 66 of age, the grower-finisher phase began and pigs were fed commercial pelleted diets in a two-phase feeding program (Table 1); the transition from grower to finisher occurred on day 105 of age. All postweaning diets were formulated to meet or exceed NRC (2012) requirements and were provided ad libitum. Subsamples for each diet were collected at the beginning of each feeding phase in each block, combined within phase, and analyzed for crude protein (AOAC, 2005; method 968.06) and calcium, phosphorus, potassium, and magnesium using inductively coupled plasma mass spectrophotometry (AOAC, 2005; method 985.01) at Agrifood Laboratories (Guelph, ON, Canada).

**Table 1.** Nutrient composition for commercial diets (as-fed basis)^1^

Item	Nursery				Grower	Finisher
	Phase I	Phase II	Phase III	Phase IV		
Calculated nutrient composition						
DE, kcal/kg	3662	3365	3401	3651	3417	3429
Crude protein, %	23.4	22.3	21.9	21.8	17.2	15.5
Ca, %	0.88	0.73	0.70	0.68	0.63	0.57
P, %	0.77	0.57	0.56	0.57	0.55	0.53
Total Lys, %	2.87	1.60	1.55	1.50	0.99	0.86
SID Lys, %^2^	1.47	1.45	1.41	1.37	0.87	0.75
SID Met, %	0.56	0.58	0.55	0.52	0.23	0.22
SID Thr, %	0.90	0.84	0.82	0.79	0.50	0.45
SID Trp, %	0.26	0.25	0.24	0.25	0.18	0.16
SID Val, %	0.95	0.87	0.85	0.84	0.62	0.54
SID Ile, %	0.90	0.81	0.78	0.76	0.51	0.43
Analyzed nutrient composition, %						
Crude protein	23.9	22.5	21.7	22.5	16.7	16.3
Ca	0.87	0.75	0.75	0.84	0.64	0.67
P	0.73	0.57	0.59	0.62	0.55	0.55
K	1.19	1.09	0.97	0.97	0.72	0.75
Mg	0.40	0.27	0.26	0.27	0.21	0.21

^1^Diets were fed after weaning for 7, 7, 7, 24, and 39 d for nursery Phases I, II, III, IV, and grower, respectively. The finisher diet was fed following the grower diet and until ~125 kg body weight. All diets were from Floradale Feedmill Ltd. (Floradale, Ontario, Canada).

^2^Standardized ileal digestible.

Individual pigs were weighed at birth and weaning, and weekly throughout the nursery period. During the grower-finisher period, body weights were recorded every other week; weekly measurements for body weight resumed as the pigs approached 100 kg BW. Per pen feed disappearance was recorded at the same times as BW for calculation of ADFI and G:F in each phase.

### Relative organ weights and gut morphology

On days 20 and 28 ± 2 of age (at weaning and 1 wk after weaning), up to 2 pigs per pen (DAY 20: parity 1–20 CON and 19 PRE; parity 2–14 CON and 22 PRE; parity 3–12 CON and 20 PRE. DAY 28: parity 1–17 CON and 22 PRE; parity 2–14 CON and 24 PRE; parity 3–12 CON and 18 PRE) were randomly selected for euthanasia, while attempting an equal sex distribution and to achieve three males and three females per pen until slaughter at market weight (~125 kg BW). Pigs were weighed and then euthanized with an intracardiac injection of pentobarbital (Euthansol; Schering Canada Inc., Pointe-Claire, QC, Canada) and exsanguination occurred via severing the major blood vessels in the neck. Liver weights were recorded. The stomach, small intestine, and large intestine were emptied, flushed with water, and weighed. On day 28 only, a 2.5 cm-segment of the jejunum (approximately 1.5 m distal to the ligament of Trietz) and ileum (approximately 0.5 m proximal to the ileo-cecal junction) were collected, rinsed with saline (0.9% sodium chloride), and placed in 10% formalin until further analysis. Following the procedures of Carleton et al. (1980), each jejunum and ileum sample was prepared for histological analysis. Using a Leica DMR fluorescence microscope (Leica Microsystems Inc., Wetzlar, Germany) and Openlab Computer Imaging System (Perkin Elmer, Waltham, MA), the average villi height and crypt depth were determined from between 6 and 10 of the longest villi from each segment.

### Carcass and loin quality evaluation

Once pigs reached ~125 kg BW (*N* = 224), up to four pigs per litter (i.e., with the goal of slaughtering the first two males and two females to reach ~125 kg BW) were transported to the University of Guelph’s Meat Laboratory. Pigs were slaughtered using CO_2_ stunning prior to death by exsanguination via severing of the major blood vessels in the neck. After slaughter, hot carcass weight was recorded; back fat and longissimus muscle (**LM**) depths were measured between the third and fourth last ribs, 7 cm from the midline on the left side of each carcass at 30 min postmortem using a Hennessy Grading Probe. The pH for the LM was measured using an Accumet A71 pH meter with a Hanna Instruments spear-tipped electrode probe attachment (Fisher Scientific, Toronto, ON) at 1 h post-mortem. The carcasses were chilled for 24 h at ≤4 °C prior to further meat quality measurements.

For up to two pigs per pen (one male and one female; *N* = 119), the left side of each carcass was weighed and then dissected into primal cuts (shoulder, belly, loin, and ham). Each primal was weighed and further dissected into lean, fat, and bone components. Prior to dissection, loins were cut into two pieces at the grading site (between the third and fourth last ribs) to expose the rib interface. Loin quality measurements were assessed by an experienced carcass evaluator. Fat depth (ruler measurement of subcutaneous fat at the grading site) and loin eye area (measured by tracing on acetate paper and quantified by an electronic planimeter; Carl Zeiss, Inc., Toronto, ON, Canada) were recorded. Five, 3-cm thick chops were then cut from the LM and trimmed of epimysium and external fat. One of the five chops was allowed to bloom for ~30 min in the Meat Lab processing room at ≤10 °C before being used to collect subjective and objective loin quality measurements. The subjective measurements of firmness (i.e. based on a three point scale; 1 = soft, to 3 = very firm), wetness (i.e., based on a three point scale; 1 = exudative, to 3 = dry), marbling (i.e. based on a ten point scale; 1 = devoid of marbling, to 10 = very abundant marbling; indicative of intramuscular fat content), and color (i.e., based on a six point scale; 1 = extremely pale pink to gray, to 6 = dark purplish red) were determined using National Pork Producers Council (NPPC 2000) procedures. Objective loin color (lightness-L*, redness-a*, and yellowness-b*) assessment was determined using a Minolta CR-400 chroma meter (Konica Minolta Sensing Americas, Inc., Ramsey, NJ) by taking the average of three measurements from different locations on the chop surface. For the 24 h postmortem pH measurement, the probe was inserted at three different locations within the chop and readings were averaged. Drip loss was measured using the methods from Honikel (1998). The remaining four chops were individually vacuum sealed and then aged at ≤4 °C for 2 or 7 d and then frozen at −20 °C until further analysis. The frozen chops were thawed over 24 h at <4 °C and used to determine cooking losses and Warner–Bratzler shear force according to the methods of Park et al. (2018).

### Statistical analysis

All data were analyzed statistically as a randomized block design using the GLIMMIX procedure of SAS. Growth performance measurements (BW, ADG, ADFI, and G:F) were analyzed for preweaning period (birthweight and ADG only), for each of the four phases during the nursery stage, and the grower and finisher periods. Litter was the experimental unit. Gestation feeding program, parity, and the interaction between gestation feeding program and parity were the main effects. Block, block by gestation feeding program, and breed within block were included as random effects. The organ data collected on days 20 and 28 ± 2 were analyzed using the same model as described above, although sex was also included as a covariate. For the number of days to reach ~125 kg BW, carcass and loin quality traits, retail cuts, and carcass components, pig was considered the experimental unit and sex was used as a covariate. For carcass and loin quality traits, retail cuts and carcass components, hot carcass weight was also used as a covariate. Preplanned contrasts were constructed to compare gestation feeding programs within parity. In each analysis, the data were considered to be significantly different when *P* < 0.05 and a trend when 0.05 ≤ *P ≤* 0.10.

## RESULTS

The main effect of parity is not mentioned in the results section; the main-effect *P-*values are presented in the tables for the reader’s information. For maternal feeding programs, the analyzed and calculated nutrient contents were comparable and sows were successfully fed according to the PRE and CON feeding programs (Stewart et al., in press). In general, the analyzed nutrient contents for the commercial diets fed to the offspring closely reflected the estimated nutrient contents (Table 1). The exceptions were nursery phase IV, where analyzed total Ca and P were ~19 and 8% greater than the calculated values and finisher, where analyzed total Ca was ~15% greater than the calculated value. Since all offspring received the same diets and growth performance was not negatively impacted, these deviations in Ca and P analyses were considered acceptable.

### Offspring growth performance

During the nursery period, there were no interactive effects of maternal gestation feeding program and parity on offspring growth performance (Table 2). As well, the main effect of maternal gestation feeding program did not influence offspring BW, ADG, ADFI, and G:F for litters during the suckling period (BW and ADG only) or for phases I to III of the nursery period (days 0 to 42). Litters from sows that received the PRE program in gestation tended to have greater BW on d 66 of age (the end of the nursery period) and greater ADG and ADFI during phase IV than litters from sows that received the CON program in gestation (main effect of maternal feeding program; *P* = 0.076, *P <* 0.05, and *P* < 0.05, for BW, ADG, and ADFI, respectively). Within parity, litters from parity 1 PRE-fed sows had greater BW on day 66 (contrast; *P* < 0.05), litters from parity 2 PRE-fed sows tended to have greater ADG in nursery phase IV (contrast; *P* = 0.075), and litters from parity 3 PRE-fed sows had greater ADFI in nursery phase IV (contrast; *P* < 0.05) vs. litters from CON-fed sows. Litter G:F was not influenced by maternal gestation feeding program during the nursery period.

**Table 2.** Offspring growth performance from sows that received either a precision (PRE) or control (CON) feeding program during gestation for three consecutive parities^1^

	Parity 1		Parity 2		Parity 3			*P*-value^2^		
	CON	PRE	CON	PRE	CON	PRE	SEM^3^	TRMT	PARITY	TRMT × PARITY
No.^4^	12	12	8	13	8	12				
Body weight, kg										
Day 0	1.36	1.29	1.41	1.53	1.50	1.61	0.11	0.609	0.166	0.657
Day 20	6.26	7.06	6.57	7.45	6.70	6.57	0.39	0.285	0.633	0.528
Day 28	7.75	8.09	8.39	8.14	7.83	7.55	0.44	0.820	0.450	0.561
Day 35	10.08	10.26	10.35	10.03	9.69	9.21	0.51	0.480	0.375	0.579
Day 42	13.4	14.1	13.9	13.6	13.4	12.7	0.6	0.821	0.497	0.275
Day 66	29.2	31.0*	28.3	29.4	28.6	28.8	1.1	0.076	0.557	0.512
Day 105	71.8	70.0	69.8	72.7†	69.1	66.6	1.7	0.655	0.150	0.050
Day 133	96.5	99.3	94.7	99.9†	101.3	96.5	2.6	0.526	0.825	0.120
Average daily gain, kg										
Birth-Wean	0.25	0.26	0.27	0.28	0.27	0.27	0.02	0.847	0.724	0.888
Phase I	0.17	0.18	0.18	0.20	0.15	0.12	0.04	0.843	0.213	0.199
Phase II	0.35	0.35	0.35	0.35	0.35	0.35	0.04	0.351	0.506	0.914
Phase III	0.52	0.59	0.51	0.52	0.53	0.51	0.05	0.384	0.776	0.310
Phase IV	0.69	0.74	0.64	0.69†	0.65	0.68	0.03	0.022	0.337	0.884
Grower	1.00	1.00	0.94	0.99*	1.01	0.93*	0.03	0.406	0.619	0.011
Finisher	1.00	1.01	1.02	1.15†	1.21	1.10†	0.06	0.780	0.041	0.045
Average daily feed intake, kg										
Phase I	0.17	0.16	0.19	0.18	0.19	0.16	0.02	0.333	0.668	0.755
Phase II	0.40	0.40	0.39	0.37	0.40	0.34	0.04	0.403	0.803	0.707
Phase III	0.64	0.68	0.66	0.68	0.65	0.63	0.06	0.543	0.832	0.712
Phase IV	1.16	1.21	1.07	1.11	1.00	1.12*	0.06	0.013	0.503	0.399
Grower	2.38	2.41	2.19	2.36	2.49	2.37	0.10	0.683	0.397	0.284
Finisher	3.14	3.17	3.13	3.58	3.37	3.18	0.19	0.494	0.670	0.159
Gain:feed										
Phase I	1.09	1.16	0.91	1.12	0.87	0.89	0.22	0.311	0.556	0.686
Phase II	0.84	0.77	0.80	0.83	0.72	0.76	0.06	0.987	0.595	0.594
Phase III	0.79	0.86	0.75	0.73	0.82	0.83	0.07	0.559	0.378	0.638
Phase IV	0.61	0.64	0.60	0.63	0.65	0.60	0.03	0.814	0.907	0.232
Grower	0.42	0.42	0.44	0.43	0.41	0.40	0.02	0.327	0.358	0.916
Finisher	0.32	0.32	0.33	0.32	0.36	0.35	0.01	0.527	0.092	0.877
No.^5^	38	40	36	60	37	56				
Days to market	162	159	162	157	153	156	3	0.248	0.126	0.145

^1^Between days 5.4 ± 2.5 and 109.7 ± 1.4 of gestation, PRE sows received unique daily blends of high and low protein diets to precisely match estimated lysine and energy requirements for individual sows. The CON sows received the same blend and quantity of high and low protein diets on each day of gestation regardless of individual sow requirements. Upon entering farrowing crates, all sows received a standard lactation diet. Sows returned to the same feeding program in each subsequent reproductive cycle.

^2^ *P*-values for the main effects of maternal feeding program in gestation (TRMT), parity (PARITY), and the interaction between maternal feeding program in gestation and parity (TRMT*PARITY).

^3^Maximum value for the standard error of the means.

^4^Number of litters evaluated; offspring were fed commercial nursery, grower, and finisher diets.

^5^Number of pigs to reach a minimum of 125 kg body weight within the experimental timeframe.

^*^Values for PRE litters or pigs are different from CON litters or pigs within parity (*P* < 0.05).

^†^Values for PRE litters or pigs tended to differ from CON litters or pigs within parity (0.05 ≤ *P* ≤ 0.10).

During the grower period, the interaction between maternal gestation feeding program and parity tended to influence BW and influenced ADG (*P =* 0.05 and *P* < 0.05, respectively; Table 2). For parity 2, litters from PRE-fed sows tended to have greater BW on day 105 (end of grower phase; contrast; *P* = 0.089; Table 2) with greater ADG (contrast; *P* < 0.05) vs. litters from CON-fed sows. Within parity 3, litters from PRE-fed sows had lower ADG (contrast; *P* < 0.05) vs. litters from CON-fed sows. There were no differences in BW or ADG between gestation feeding programs in parity 1. Litter ADFI and G:F were not influenced by the main effect of maternal gestation feeding program during the grower period.

During the finisher period, litters from PRE-fed sows tended to have greater BW on day 133 of age within parity 2 only (contrast; *P* = 0.090; Table 2) vs. litters from CON-fed sows. A maternal gestation feeding program by parity interaction influenced ADG (*P* < 0.05) such that for parity 2, litters from PRE-fed sows tended to have greater ADG (contrast; *P* = 0.054) while for parity 3, litters from PRE-fed sows tended to have lower ADG (contrast; *P* = 0.099) vs. litters from CON-fed sows. Otherwise, ADFI and G:F in the finisher phase, and the number of days to reach market weight (~125 kg BW) were not influenced by the main effect of maternal gestation feeding program or the interaction between gestation feeding program and parity.

### Relative organ weights and gut morphology

At weaning (day 20 ± 2), there were no interactive effects of maternal feeding program and parity on offspring live BW or relative organ weights (% of BW; Table 3). Offspring live BW and relative organ weights were also not influenced by the main effect of maternal feeding program, with the exception that offspring from PRE-fed sows tended to have lower relative large intestine weights (*P* = 0.097) than offspring from CON-fed sows. One-week after weaning (day 28 ± 2), the interaction between maternal gestation feeding program and parity influenced the relative weights of liver (tendency; *P* = 0.097), small intestine (*P* < 0.05), and gastrointestinal tract (**GIT**; *P* < 0.05). In parity 3 only, the relative weights of the small intestine and GIT were and tended to be less (contrast; *P* < 0.05 and *P* = 0.073, respectively) for the offspring from PRE-fed sows vs. the offspring from CON-fed sows. In parity 1 only, the relative large intestine weight was greater (contrast; *P* < 0.05) for the offspring from PRE-fed sows vs. the offspring from CON-fed sows. There were no main or interactive effects of maternal feeding program and parity on ileum and jejunum villi height, crypt depth, and villus:crypt ratio 1 wk after weaning (Table 4).

**Table 3.** Relative organ weights for pigs at 20 and 28 d of age from sows that received either a precision (PRE) or control (CON) feeding program during gestation for three consecutive parities^1^

	Parity 1		Parity 2		Parity 3			P-value^2^		
	CON	PRE	CON	PRE	CON	PRE	SEM^3^	TRMT	PARITY	TRMT × PARITY
Day 20										
No.^4^	20	19	14	22	12	20				
Live body weight, kg	5.91	6.21	6.80	6.70	6.58	6.96	0.51	0.637	0.353	0.843
Liver, % BW	2.43	2.60	2.46	2.44	2.45	2.46	0.12	0.598	0.849	0.668
Stomach, % BW	0.59	0.56	0.51	0.55	0.52	0.52	0.02	0.685	0.055	0.172
Small intestine, % BW	3.68	3.78	3.72	3.65	3.70	3.70	0.22	0.935	0.967	0.888
Large intestine, % BW	1.07	0.99	0.92	0.90	0.98	0.88	0.07	0.097	0.298	0.636
Gastrointestinal tract (GIT), % BW	5.30	5.44	5.09	5.09	5.15	5.11	0.28	0.876	0.471	0.924
Day 28										
No.^5^	17	22	15	24	12	18				
Live body weight, kg	7.12	7.46	7.34	7.92	7.06	7.86	0.46	0.107	0.775	0.791
Liver, % BW	2.25	2.51	2.31	2.44	2.43	2.25	0.14	0.357	0.949	0.097
Stomach, % BW	0.71	0.67	0.68	0.70	0.71	0.64	0.05	0.397	0.840	0.415
Small intestine, % BW	4.35	4.66	4.13	4.34	4.64	4.07*	0.37	0.909	0.806	0.040
Large intestine, % BW	1.43	1.64*	1.58	1.66	1.47	1.41	0.14	0.148	0.364	0.137
GIT, % BW	6.46	6.98	6.41	6.70	6.80	6.11†	0.52	0.843	0.906	0.042

^1^Between days 5.4 ± 2.5 and 109.7 ± 1.4 of gestation, PRE sows received unique daily blends of high and low protein diets to precisely match estimated lysine and energy requirements for individual sows. The CON sows received the same blend and quantity of high and low protein diets on each day of gestation regardless of individual sow requirements. Upon entering farrowing crates, all sows received a standard lactation diet. Sows returned to the same feeding program in each subsequent reproductive cycle. All gastrointestinal organs were emptied of digesta prior to weighing.

^2^ *P*-values for the main effects of maternal feeding program in gestation (TRMT), parity (PARITY), and the interaction between maternal feeding program in gestation and parity (TRMT × PARITY).

^3^Maximum value for the standard error of the means.

^4^Number of piglets evaluated at 20 d of age; randomly selected from 12 CON and 12 PRE litters (parity 1), 8 CON and 12 PRE litters (parity 2), and 7 CON and 10 PRE litters (parity 3).

^5^Number of piglets evaluated at 28 d of age; randomly selected from 9 CON and 12 PRE litters (parity 1), 8 CON and 13 PRE litters (parity 2), and 8 CON and 10 PRE litters (parity 3).

^*^Values for PRE litters or pigs are different from CON litters or pigs within parity (*P* < 0.05).

^†^Values for PRE litters or pigs tended to differ from CON litters or pigs within parity (0.05 ≤ *P* ≤ 0.10).

**Table 4.** Offspring jejunal and ileal morphology at 28 d of age from sows that received either a precision (PRE) or control (CON) feeding program during gestation for three consecutive parities^1^

	Parity 1		Parity 2		Parity 3			*P*-value^2^		
	CON	PRE	CON	PRE	CON	PRE	SEM^3^	TRMT	PARITY	TRMT × PARITY
No.^4^	17	22	15	24	12	16				
Jejunum morphology, μm										
Villus height	349	412	399	344	385	394	28	0.796	0.706	0.134
Crypt depth	155	157	155	141	141	150	13	0.888	0.706	0.497
Villus:crypt ratio	2.40	2.69	2.55	2.44	2.93	2.66	0.27	0.861	0.304	0.405
Ileum morphology, μm										
Villus height	364	371	358	388	387	398	32	0.460	0.695	0.870
Crypt depth	155	157	159	161	153	151	10	0.962	0.646	0.969
Villus:crypt ratio	2.47	2.35	2.32	2.43	2.55	2.74	0.15	0.588	0.164	0.489

^1^Between days 5.4 ± 2.5 and 109.7 ± 1.4 of gestation, PRE sows received unique daily blends of high and low protein diets to precisely match estimated lysine and energy requirements for individual sows. The CON sows received the same blend and quantity of high and low protein diets on each day of gestation regardless of individual sow requirements. Upon entering farrowing crates, all sows received a standard lactation diet. Sows returned to the same feeding program in each subsequent reproductive cycle.

^2^ *P*-values for the main effects of maternal feeding program in gestation (TRMT), parity (PARITY), and the interaction between maternal feeding program in gestation and parity (TRMT × PARITY).

^3^Maximum value for the standard error of the means.

^4^Number of ileum and jejunum samples obtained from piglets at 28 d of age; randomly selected from 9 CON and 12 PRE litters (parity 1), 8 CON and 13 PRE litters (parity 2), and 8 CON and 10 PRE litters (parity 3).

^*^Values for PRE litters or pigs are different from CON litters or pigs within parity (*P* < 0.05).

^†^Values for PRE litters or pigs tended to differ from CON litters or pigs within parity (0.05 ≤ *P* ≤ 0.10).

### Carcass and loin quality at market weight

For the pigs slaughtered at ~125 kg BW, there were no interactive effects of maternal gestation feeding program and parity on the carcass quality traits analyzed (Table 5). The main effect of maternal gestation feeding program also did not affect carcass quality traits at slaughter. In parity 2 only, fat depth tended to be greater (contrast; *P* = 0.055) and lean yield tended to be less (contrast; *P* = 0.051) for offspring from PRE-fed sows vs. offspring from CON-fed sows. Also in parity 2, the 1-hour postmortem pH values for the loin tended to be greater (contrast; *P* = 0.070) while the 24-h loin pH tended to be less (contrast; *P* = 0.080) for offspring from PRE-fed vs. CON-fed sows (Table 5).

**Table 5.** Carcass quality characteristics at market weight (~125 kg BW) for offspring from sows that received either a precision (PRE) or control (CON) feeding program during gestation for three consecutive parities^1^

	Parity 1		Parity 2		Parity 3			*P*-value^2^		
	CON	PRE	CON	PRE	CON	PRE	SEM^3^	TRMT	PARITY	TRMT × PARITY
No.^4^	42	41	25	43	29	44				
Slaughter weight, kg	126.4	127.1	128.8	129.7	130.6	130.5	1.0	0.365	0.024	0.700
Hot carcass weight, kg	103.6	104.1	105.0	105.2	105.9	106.2	1.1	0.409	0.228	0.951
Probe fat depth, mm	20.2	21.4	20.4	22.7†	22.2	21.3	0.9	0.182	0.410	0.182
Probe lean depth, mm	58.1	56.8	57.8	57.0	56.2	55.6	1.8	0.482	0.717	0.973
Lean yield, %	59.7	59.2	59.6	58.6†	58.8	59.2	0.4	0.196	0.445	0.175
LM pH in loin, 1 h postmortem^5^	6.48	6.51	6.45	6.58†	6.60	6.65	0.11	0.123	0.455	0.570
LM pH in loin, 24 h postmortem	5.64	5.60	5.67	5.57†	5.61	5.58	0.04	0.130	0.747	0.577

^1^Between days 5.4 ± 2.5 and 109.7 ± 1.4 of gestation, PRE sows received unique daily blends of high and low protein diets to precisely match estimated lysine and energy requirements for individual sows. The CON sows received the same blend and quantity of high and low protein diets on each day of gestation regardless of individual sow requirements. Upon entering farrowing crates, all sows received a standard lactation diet. Sows returned to the same feeding program in each subsequent reproductive cycle.

^2^ *P*-values for the main effects of maternal feeding program in gestation (TRMT), parity (PARITY), and the interaction between maternal feeding program in gestation and parity (TRMT*PARITY).

^3^Maximum value for the standard error of the means.

^4^Number of pigs evaluated after slaughter at approximately 125 kg body weight.

^5^LM – longissimus.

^*^Values for PRE litters or pigs are different from CON litters or pigs within parity (*P* < 0.05).

^†^Values for PRE litters or pigs tended to differ from CON litters or pigs within parity (0.05 ≤ *P* ≤ 0.10).

There were no interactive effects of maternal gestation feeding program and parity on loin quality traits (Table 6). Loin eye area was smaller (main effect of maternal feeding program; *P* < 0.01) for offspring from PRE-fed vs. CON-fed sows, which was especially evident in parity 3 (contrast; *P* < 0.05; Table 6). Other loin quality traits including firmness, wetness, marbling, subjective and objective color, drip loss, shear force, and cooking losses were not influenced by maternal gestation feeding program.

**Table 6.** Subjective and objective loin characteristics at market weight (~125 kg BW) for offspring from sows that received either a precision (PRE) or control (CON) feeding program during gestation for three consecutive parities^1^

	Parity 1		Parity 2		Parity 3			*P*-value^2^		
	CON	PRE	CON	PRE	CON	PRE	SEM^3^	TRMT	PARITY	TRMT × PARITY
No.^4^	42	41	25	43	29	44				
Fat ruler measure, mm^5^	16.8	17.8	17.5	18.8	19.4	19.2	0.97	0.303	0.055	0.623
LM area, cm^2^^6^	55.8	53.9	55.3	52.9	53.8	49.7*	1.8	0.007	0.319	0.667
Firmness, scale from 1 to 3^7^	1.9	1.7	2.0	2.1	2.0	1.9	0.1	0.179	0.096	0.454
Wetness, scale from 1 to 3^8^	2.0	2.3	2.0	2.0	2.0	2.1	0.12	0.162	0.590	0.348
Marbling, scale from 1 to 10^9^	1.7	1.7	2.5	2.5	2.8	2.3	0.26	0.358	0.033	0.403
Color, scale from 1 to 6^10^	3.1	3.1	3.2	3.2	3.3	3.4	0.14	0.583	0.327	0.991
L*^11^	47.4	47.2	47.8	48.2	48.3	47.8	0.94	0.848	0.771	0.701
a*	6.7	6.5	7.0	6.5	6.7	6.8	0.27	0.225	0.757	0.431
b*	3.2	3.3	3.5	3.3	3.0	2.8	0.39	0.760	0.294	0.937
Drip loss, %	2.39	2.34	1.74	1.86	1.92	1.56	0.36	0.730	0.176	0.737
Cooking losses 2 d aged, %	22.34	21.45	21.94	22.33	23.03	23.64	0.89	0.949	0.284	0.608
Cooking losses 7 d aged, %	22.55	22.99	24.14	24.13	23.35	23.96	0.94	0.602	0.391	0.906
Shear force 2 d aged, kg	4.95	4.83	5.05	4.50	4.76	4.65	0.34	0.352	0.853	0.751
Shear force 7 d aged, kg	4.41	4.26	4.99	4.07	4.73	4.35	0.49	0.218	0.888	0.650

^1^Between days 5.4 ± 2.5 and 109.7 ± 1.4 of gestation, PRE sows received unique daily blends of high and low protein diets to precisely match estimated lysine and energy requirements for individual sows. The CON sows received the same blend and quantity of high and low protein diets on each day of gestation regardless of individual sow requirements. Upon entering farrowing crates, all sows received a standard lactation diet. Sows returned to the same feeding program in each subsequent reproductive cycle.

^2^ *P*-values for the main effects of maternal feeding program in gestation (TRMT), parity (PARITY), and the interaction between maternal feeding program in gestation and parity (TRMT × PARITY).

^3^Maximum value for the standard error of the means.

^4^Number of pigs evaluated after slaughter at approximately 125 kg body weight.

^5^Ruler measurement of subcutaneous fat at the grading site.

^6^LM – longissimus.

^7^National Pork Producers Council (NPPC, 2000): 1 = soft – cut surfaces distort easily and are visibly soft, 2 = firm – cut surfaces tend to hold their shape, 3 = very firm – cut surfaces tend to be very smooth with no distortion of shape.

^8^National Pork Producers Council (NPPC, 2000): 1 = exudative – excessive fluid pooling on cut surfaces, 2 = moist – cut surfaces appear moist, with little or no free water, 3 = dry – cut surfaces exhibit no evidence of free water.

^9^National Pork Producers Council (NPPC, 2000): 1 = devoid of marbling, to 10 = very abundant marbling.

^10^National Pork Producers Council (NPPC, 2000): 1 = pale pinkish gray to white, 2 = grayish pink, 3 = reddish pink, 4 = dark reddish pink, 5 = purplish red, 6 = dark purplish red.

^11^L* Lightness, a* redness, b* yellowness.

^*^Values for PRE litters or pigs are different from CON litters or pigs within parity (*P* < 0.05).

^†^Values for PRE litters or pigs tended to differ from CON litters or pigs within parity (0.05 ≤ *P* ≤ 0.10).

For the carcasses that were dissected into primal cuts, the interaction between maternal gestation feeding program and parity tended to influence relative tenderloin weight (% of left side carcass weight; *P* = 0.070; Table 7). Over all three parities and particularly in parity 3, pigs from PRE-fed sows had greater (*P* < 0.05 and *P* = 0.022 for the main effect of maternal feeding program and parity 3 contrast, respectively) relative tenderloin weights vs. the pigs from CON-fed sows. Otherwise, carcass weight, and retail cuts and components were not influenced by maternal gestation feeding program.

**Table 7.** Relative weights for retail cuts at market weight (~125 kg BW) for offspring from sows that received either a precision (PRE) or control (CON) feeding program during gestation for three consecutive parities^1^

	Parity 1		Parity 2		Parity 3			*P*-value^2^		
	CON	PRE	CON	PRE	CON	PRE	SEM^3^	TRMT	PARITY	TRMT × PARITY
No.^4^	23	21	14	22	15	24				
Left side carcass wt, kg	47.0	47.7	47.7	47.5	47.8	47.7	0.3	0.595	0.361	0.202
Shoulder, primal, %^5^	19.4	18.8	19.0	19.1	19.3	19.6	0.5	0.691	0.636	0.307
Butt, retail, %	9.1	8.9	8.7	9.2	8.8	8.9	0.3	0.564	0.896	0.519
Picnic, retail, %	10.5	10.0	9.6	10.3	10.4	10.5	0.3	0.654	0.193	0.171
Belly, primal, %	18.4	18.1	18.9	18.8	18.8	18.7	0.7	0.577	0.726	0.994
Belly, retail, %	9.1	9.4	9.1	9.3	9.6	9.7	0.6	0.628	0.738	0.976
Loin, primal, %	27.1	27.5	28.2	27.8	27.6	27.5	0.8	0.925	0.724	0.854
Tenderloin, %	1.0	1.1	1.1	1.1	0.9	1.1*	0.1	0.016	0.716	0.070
Ham, primal, %	24.9	25.0	24.6	24.4	23.8	23.2	0.8	0.508	0.296	0.764
Ham, retail, %	18.8	18.8	18.3	17.8	17.9	17.8	0.6	0.664	0.459	0.879
Bone, %	7.9	8.3	8.7	8.0	8.1	8.1	0.3	0.818	0.632	0.328
Fat trim, %	15.7	15.5	14.3	16.8	15.8	15.8	1.5	0.398	0.976	0.535
Lean trim, %	4.9	4.9	3.9	3.9	3.7	4.0	0.3	0.650	0.003	0.688

^1^Between days 5.4 ± 2.5 and 109.7 ± 1.4 of gestation, PRE sows received unique daily blends of high and low protein diets to precisely match estimated lysine and energy requirements for individual sows. The CON sows received the same blend and quantity of high and low protein diets on each day of gestation regardless of individual sow requirements. Upon entering farrowing crates, all sows received a standard lactation diet. Sows returned to the same feeding program in each subsequent reproductive cycle.

^2^ *P*-values for the main effects of maternal feeding program in gestation (TRMT), parity (PARITY), and the interaction between maternal feeding program in gestation and parity (TRMT × PARITY).

^3^Maximum value for the standard error of the means.

^4^Number of pigs evaluated 24 h after slaughter.

^5^All cuts expressed as a percentage of the left side carcass weight.

^*^Values for PRE litters or pigs are different from CON litters or pigs within parity (*P* < 0.05).

^†^Values for PRE litters or pigs tended to differ from CON litters or pigs within parity (0.05 ≤ *P* ≤ 0.10).

## DISCUSSION

The objective of the current study was to determine the effects of closely meeting the estimated daily energy and Lys requirements for gestating sows over three consecutive parities on offspring postweaning growth performance and carcass characteristics and loin quality at market weight (~125 kg BW). The ADG and ADFI during the final phase of the nursery period and nursery exit BW were greater for offspring from PRE- vs. CON-fed sows (main effect of maternal feeding program). On average, offspring from PRE-fed sows were 1 kg heavier at the end of the nursery period vs. offspring from CON-fed sows. The greater ADG in nursery phase four and nursery exit BW for offspring from PRE-fed sows was driven by greater ADFI and not an improvement in nutrient utilization efficiency, since G:F was not affected. Previous research has demonstrated an improvement in growth performance (ADG, ADFI, and G:F) during the growing and finishing periods, fewer days to market, and greater carcass yield when greater ADG and exit BW were achieved in the nursery period (Kim et al., 2001; Wolter and Ellis, 2001). In the current study, the average 1-kg BW advantage was still present on day 133 of age (end of finisher period) for the offspring from PRE-fed sows, but was not significant at heavier BW. Although not statistically significant, offspring from PRE-fed sows achieved the market weight of 125 kg BW ~4 d earlier in parities 1 and 2 vs. the offspring from CON-fed sows, which seems to indicate an overall advantage of precision feeding during gestation on offspring productivity when the sows are still immature. This observation could be attributed to more precisely matching the estimated daily Lys and energy requirements for younger sows, since these sows have requirements for maternal growth in addition to maintenance and pregnancy-associated protein and energy deposition during gestation (NRC, 2012; Kim et al., 2013; Thomas et al., 2018), which could benefit fetal development and subsequent postweaning growth performance. By the third parity however, the litters from PRE-fed sows no longer showed numerically superior growth performance in the nursery or days to market, which could indicate that older sows are less sensitive to the apparent benefits of precision feeding during gestation, resulting in fewer advantages for the offspring. Therefore, precision feeding during gestation may be more favorable for herds with a greater proportion of young sows in terms of post-weaning offspring growth performance.

The mechanism driving improved ADG in nursery phase IV and nursery exit BW for offspring from PRE-fed sows remains to be elucidated. The greater ADG, ADFI, and nursery exit BW for offspring from PRE-fed sows did not correspond to larger relative gastrointestinal organ weights (days 20 and 28 of age) or improved jejunal or ileal histomorphology 1 wk after weaning (day 28 of age). Though, it is noted that these measurements were collected at least 2 wk prior to when differences were observed for ADG, ADFI, and nursery exit BW, and may not represent gastrointestinal tract capacity during nursery phase IV. Other studies have shown that improved ADG was related to a pig’s ability to consume feed and digest and absorb nutrients after weaning, which corresponded to a heavier and more developed small intestine (Pluske et al., 1997). Moreover, others have shown that differences in offspring intestinal size and morphology can be influenced by maternal diet during gestation (Draper et al., 2016; Chen et al., 2017). For example, Chen et al. (2017) found that offspring from sows that were energy-restricted throughout gestation had lower relative small intestine weights combined with smaller villus height and crypt depth and lower BW at 28 of age. Others have also demonstrated that an insufficient supply of dietary energy in gestation adversely affected fetal gastrointestinal tract maturity at birth and at 28 d of age (Cao et al., 2014; Liu et al., 2016). In the present study, the CON feeding program supplied energy below estimated requirements, particularly during the second half of gestation for parity 1 and 2 sows (NRC, 2012; Stewart et al., in press). Determining whether the maternal feeding programs influenced the development of the fetal gastrointestinal tract was beyond the scope of the current study, but piglet birth weight, as well as, ADG during the suckling phase and BW at weaning were unaffected by maternal feeding program (Stewart et al., in press). Therefore, it is possible that the relative gastrointestinal organ weights diverged later in the nursery period to accommodate greater ADFI during nursery phase IV for offspring from sows that received the PRE feeding program during gestation. However, organ characteristics were not measured at the end of the nursery period and differences in ADG and ADFI did not continue into the growing and finishing phases.

In the current study, maternal feeding program generally did not influence the carcass or loin quality for the offspring. In North America, carcass value is determined based on hot carcass weight and lean yield percentage (Heyer et al., 2004). For example, the OlyWest H 2021 Wide Window Grid rewards pigs with hot carcass weights between 103.0 and 107.9 kg and lean yield values between 58.60% and 61.79% with the highest index. In the current study, the pigs sent to market typically achieved both of these criteria for a premium index, despite offspring from PRE-fed sows in parity 2 having slightly lower carcass yield (58.6% vs. 59.6%) and an overall reduction in LM area (52.2 vs. 55.0 cm^2^) than offspring from CON-fed sows. Moreover, there were generally no differences for the relative proportions of retail cuts between offspring from the two gestation feeding programs throughout all three parities. It was expected that improvements in ADG would correspond to greater lean yields. However, ADG was only greater for offspring from PRE-fed sows during nursery phase IV, with no further differences in the grower and finisher phases. It is probable that the ADG during the grower and finisher phases has a greater impact on carcass lean yield vs. ADG during the nursery phase. Finally, loin quality characteristics (e.g., color, marbling, drip loss, cooking loss, and shear force) were not different between offspring from sows that received the PRE or CON feeding programs during gestation, indicating that there were no improvements or detriments to meat quality and likely, the eating experience for consumers. Therefore, achieving the same carcass value and considering 4 fewer days to market for offspring from PRE-fed parity 1 and 2 sows, there may be opportunity to elicit feed cost and yardage savings, as well as faster turnaround times for nursery facilities when raising offspring from sows that received a PRE feeding program in gestation. Though, the observations noted in the current study should be confirmed in a larger, commercial-scale environment.

## CONCLUSION

Precisely meeting estimated daily Lys and energy requirements during gestation throughout three consecutive parities improved offspring ADG and ADFI in the final phase of the nursery period, but this was not related to improvements in feed efficiency, relative gastrointestinal organ weights, or small intestinal histomorphology. Precision feeding Lys and energy during gestation had minimal effects on offspring growth performance during the grower and finisher phases, or carcass and loin quality at slaughter. Since there were no differences in offspring feed efficiency or carcass value, the potential for economic benefit from using a precision feeding program for gestating sows may be achieved with four fewer days to market for offspring from parity 1 and 2 sows leading to reduced feed costs, yardage savings, and a faster barn turnover time. Future research should evaluate similar feeding programs for gestating sows in commercial environments.

## ABBREVIATIONS

CON: control
ESF: electronic sow feeder
GIT: gastrointestinal tract
LM: longissimus
NPPC: National Pork Producers Council
PRE: precision
SID: standard ileal digestibility
